# Supplementary material for: Ubiquitin-Specific Proteases as Potential Therapeutic Targets in Bladder Cancer—In Vitro Evaluation of Degrasyn and PR-619 Activity Using Human and Canine Models
Source: Biomedicines. 2023 Mar 2;11(3):759. doi: 10.3390/biomedicines11030759 (PMC10045593; doi:10.3390/biomedicines11030759)
Supplement: Supplementary file 1 [file biomedicines-11-00759-s001.zip › biomedicines-2092404-supplementary.pdf]

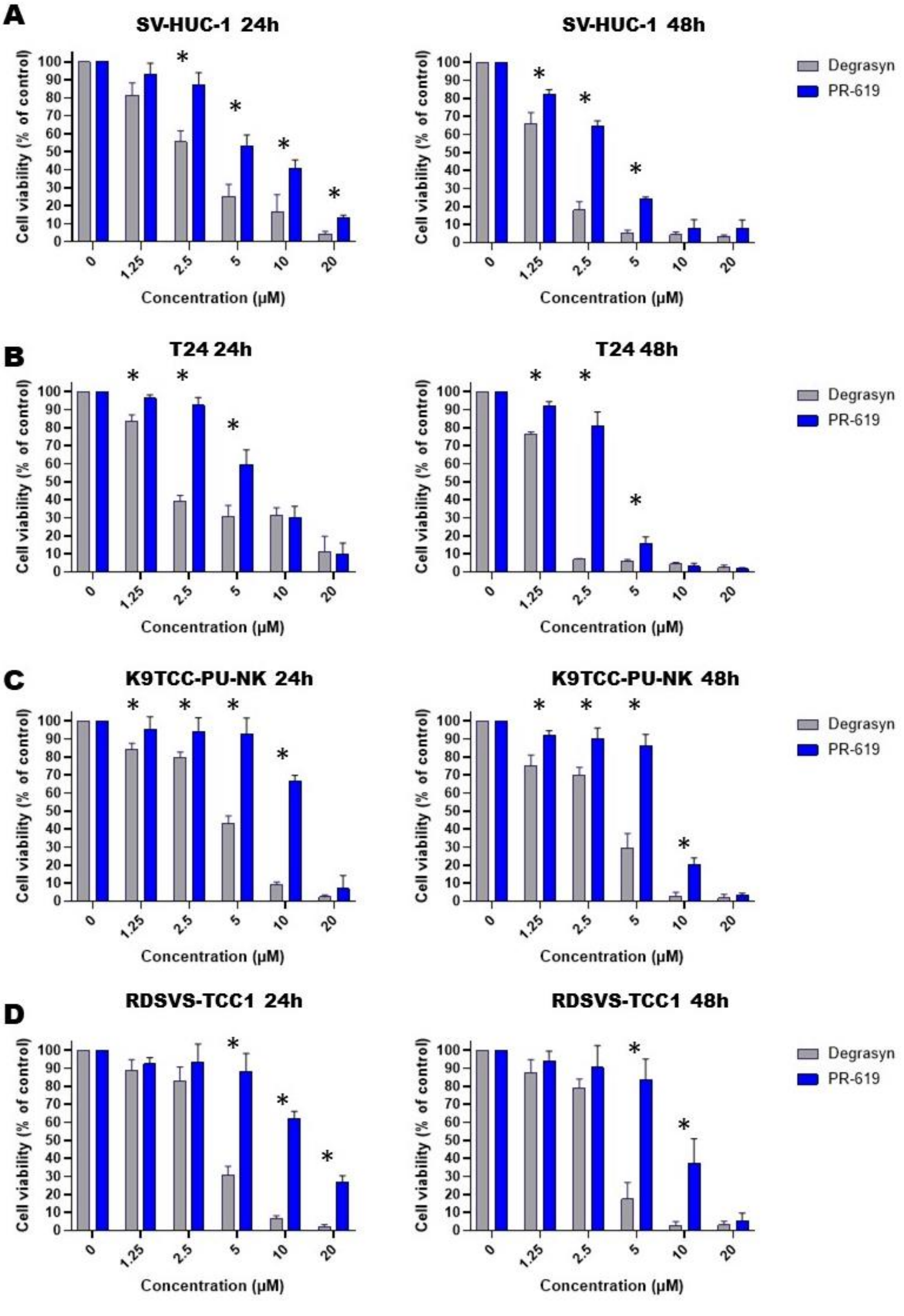

Supplementary Figure 1. Comparison of Degrasyn and PR-619 influence on the cell proliferation of SV-HUC-1 (**A**), T24 (**B**), K9TCC-PU-NK (**C**), and RDSVS-TCC1 (**D**) cell lines (MTT assay) after 24 and 48 h with increasing concentrations of the drugs (data for 1.25, 2.5, 5, 10, and 20 μM). Values are presented as means ± standard deviations (SDs) of three independent experiments. * Considered significant in comparison to control ($p < 0.05$).

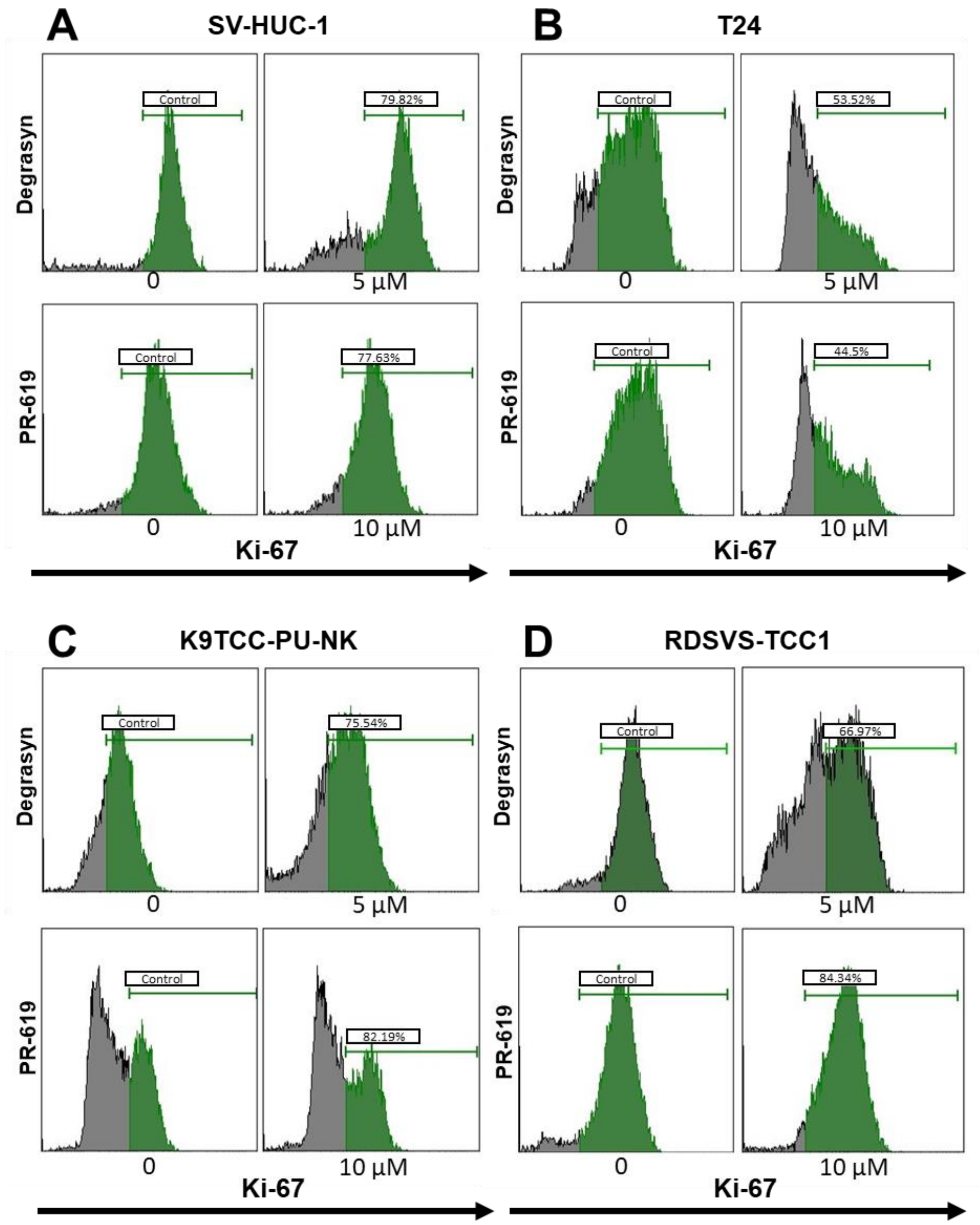

Supplementary Figure 2. Ki-67 flow cytometry proliferation assay. Representative histograms showing the percentage of Ki-67 positive cells (in reference to control) after 24 h exposure to increasing concentrations of Degrasyn and PR-619 in (**A**) SV-HUC-1, (**B**) T24, (**C**) K9TCC-PU-NK, and (**D**) RDSVS-TCC cell lines.
